# Roles of two distinct alphasatellites modulating geminivirus pathogenesis

**DOI:** 10.1186/s12985-021-01718-6

**Published:** 2021-12-13

**Authors:** Manish Kumar, Fauzia Zarreen, Supriya Chakraborty

**Affiliations:** Molecular Virology Laboratory, School of Life Sciences, Jawaharlal Nehru University, New Delhi, 110 067 India

**Keywords:** Geminivirus, Begomovirus, Alphasatellite, Betasatellite, Genetic variability, Disease development

## Abstract

**Background:**

Alphasatellites are small coding DNA satellites frequently associated with a begomovirus/betasatellite complex, where they are known to modulate virulence and symptom development. Two distinct alphasatellites, namely, Cotton leaf curl Multan alphasatellite (CLCuMuA), and Gossypium darwinii symptomless alphasatellite (GDarSLA) associated with Cotton leaf curl Multan virus-India (CLCuMuV-IN) and Ludwigia leaf distortion betasatellite (LuLDB) were found to be associated with yellow mosaic disease of hollyhock (*Alcea rosea*) plants. In this study, we show that alphasatellites CLCuMuA and GDarSLA attenuate and delay symptom development in *Nicotiana benthamiana*. The presence of either alphasatellites reduce the accumulation of the helper virus CLCuMuV-IN. However, the levels of the associated betasatellite, LuLDB, remains unchanged. These results suggest that the alphasatellites could contribute to the host defence and understanding their role in disease development is important for developing resistance strategies.

**Methods:**

Tandem repeat constructs of two distinct alphasatellites, namely, CLCuMuA and GDarSLA associated with CLCuMuV-IN and LuLDB were generated. *N. benthamiana* plants were co-agroinoculated with CLCuMuV and its associated alphasatellites and betasatellite molecules and samples were collected at 7, 14 and 21 days post inoculation (dpi). The viral DNA molecules were quantified in *N. benthamiana* plants by qPCR. The sequences were analysed using the MEGA-X tool, and a phylogenetic tree was generated. Genetic diversity among the CLCuMuA and GDarSLA was analysed using the DnaSP tool.

**Results:**

We observed a reduction in symptom severity and accumulation of helper virus in the presence of two alphasatellites isolated from naturally infected hollyhock plants. However, no reduction in the accumulation of betasatellite was observed. The phylogenetic and genetic variability study revealed the evolutionary dynamics of these distinct alphasatellites , which could explain the role of hollyhock-associated alphasatellites in plants.

**Conclusions:**

This study provides evidence that alphasatellites have a role in symptom modulation and suppress helper virus replication without any discernible effect on the replication of the associated betasatellite.

**Supplementary Information:**

The online version contains supplementary material available at 10.1186/s12985-021-01718-6.

## Background

Viruses of the genus *Begomovirus*, family *Geminiviridae* have a small circular ssDNA genome and are transmitted by whiteflies [1]. The genome of begomoviruses are bipartite (components known as DNA A and DNA B) or monopartite (a single component similar to DNA A of abipartite begomovirus) [1]. At least three classes of circular ssDNA satellites associated with begomoviruses have been described: betasatellites [2], alphasatellites [3] and deltasatellites [4, 5].

Betasatellites (1.5–1.7 kb) genome contain a satellite conserved region (SCR) and a single gene, βC1. Betasatellites depend on the helper begomovirus for their replication, movement and transmission. βC1 is known to be a symptom determinant and thus, enhances the symptoms in some pathosystems [6–8]. It is also known to suppress transcriptional (TGS) and post-transcriptional gene silencing (PTGS) [7, 9, 10].

Alphasatellites (1.5–1.7 kb) associate with the begomovirus/betasatellite complex and depend on the helper begomovirus only for their movement in plants and vector transmission. They encode their own nanovirus-like replication initiator protein (Alpha-Rep) which enables them to replicate autonomously in plant cells [11, 12]. The Rep of some alphasatellites is known to suppress PTGS and TGS [13, 14]. Alphasatellites have a role in pathogenicity as well. At least two alphasatellites have been shown to attenuate disease symptoms and reduce the accumulation of the helper begomovirus and its associated betasatellite [15, 16]. In contrast, the association of alphasatellites with *Wheat dwarf India virus* (WDIV), a mastrevirus, increases WDIV accumulation and reduces viral small interfering RNA (VsiRNA) levels [17].

Previously, we have reported the presence of an isolate of monopartite *Cotton leaf curl Multan virus-India* (CLCuMuV-IN, Accession no. MG373551) and *Ludwigia leaf distortion betasatellite* (LuLDB, Accession no. MG373553) from hollyhock (*Alcea rosea*) plants exhibiting yellow mosaic symptoms [18]. Further, we isolated two alphasatellites *Cotton leaf curl Multan alphasatellite* (CLCuMuA, Accession no. MG373558) and *Gossypium darwinii symptomless alphasatellite* (GDarSLA, Accession no. MG373559) from the same hollyhock plants [18]. In this study, we demonstrate the role of CLCuMuA and GDarSLA in pathogenicity and more specifically their ability to delay and attenuate symptom severity and reduce the accumulation of the helper virus CLCuMuV-IN, with no apparent effect on the accumulation of the associated betasatellite LuLDB.

## Methods

### Construction of agroinfectious clones

**Fig. 1:**
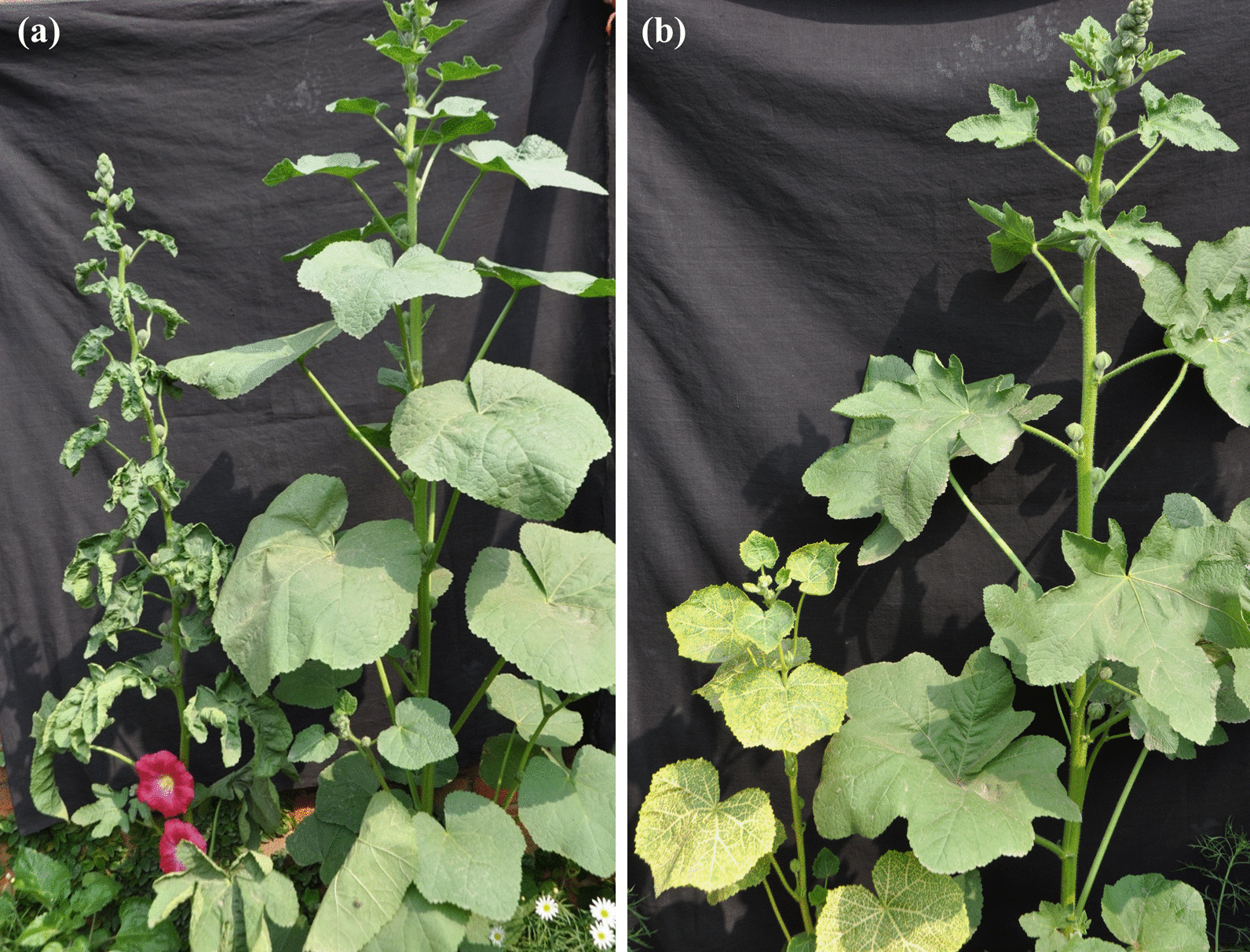
Symptoms induced by the begomoviruses on hollyhock (*A. rosea*) plants showing **a** leaf curling and **b** yellowing of leaves inthe field condition. Non-symptomatic or healthy plants were also shown adjacent to the diseased one for the identification of the diseased plants

Hollyhock plants exhibiting typical leaf curling symptoms, veinal chlorosis, vein thickening, and yellow mosaic symptoms (Fig. 1) were collected and various geminivirus DNAs were previously cloned from these samples [18]. These clones available in our laboratory were used for generation of infectious clones. Partial tandem repeat constructs (PTR) of *Cotton leaf curl Multan virus-India* (CLCuMuV-IN, Accession no. MG373551) and *Ludwigia leaf distortion betasatellite* (LuLDB, Accession no. MG373553) and two alphasatellites *Cotton leaf curl Multan alphasatellite* (CLCuMuA, Accession no. MG373558) and *Gossypium darwinii symptomless alphasatellite* (GDarSLA, Accession no. MG373559) were generated in this study. For CLCuMuV-IN, the *Eco*RV (300 nt)-*Xho*I (2077 nt) fragment containing the CR region was cloned into the pBluescript II KS (+) vector, followed by the monomeric DNA-A molecules to generate the PTR construct. Similarly, for CLCuMuA alphasatellite, approximately 1.2 kb *Eco*RI (515 nt)-*BamH*I (1526 nt) fragment and for GDarSLA, *EcoR*I (508 nt)-*Sal*I (1355 nt) fragment of the satellite DNA was digested and cloned into the pBluescript II KS (+) vector and further mobilized into the binary vector pCAMBIA2300 at *Xba*I and *Kpn*I site. For LuLDB, the 500 bp of *Kpn*I (507 nt)-*Sal*I (1307 nt) fragment was cloned in pCAMBIA2300 followed by ligation of the full-length *Kpn*I digested monomer fragment to generate the PTR constructs of betasatellite [19, 20]. Further, these infectious clones were transformed into the *Agrobacterium* EHA105 for infectivity analysis in *N. benthamiana* plants.

### Agrobacterium-mediated inoculation and infectivity analysis

*N. benthamiana* wild-type plants were grown in an insect-free growth chamber at 25 ± 2 °C, 60–70% relative humidity, and 16/8 h (light/dark) photoperiod. *Agrobacterium* *tumefaciencs* strain EHA105 cultures harbouring agroinfectious clones of CLCuMuV-IN, LuLDB, CLCuMuA and GDarSLA were grown overnight at 28 °C in Luria–Bertani broth supplemented with 50 µgml^−1^ kanamycin. *Agrobacterium* cultures were mixed in equal ratio  and *N. benthamiana* plants were inoculated at the four leaves stage as described by [21]. The inoculated plants were monitored for the appearance of symptoms under insect-proof controlled conditions for 30 days. *A. tumefaciencs* starin EHA105 harbouring the vector pCAMBIA2300 was used for mock inoculation. The agroinoculated plants were regularly scored for symptoms on the scale of 0 to 5 for 30dpi [22]. Symptom severity scale, 0-no symptoms, 1-very mild yellowing, 2-mild yellowing with downward leaf curling, 3-severe yellowing of leaves with downward leaf curling and vein thickening, 4-severe yellowing of leaves with downward leaf curling, vein thickening and stunted plant growth, 5-severe yellowing of leaves with downward leaf curling, vein thickening and stunted plant growth leading to plant death. The uppermost leaves of inoculated *N. benthamiana* plants were collected at 7, 14 and 21 dpi. The presence of CLCuMuV-IN, LuLDB, CLCuMuA and GDarSLA was checked by PCR. The infectivity analysis was repeated a minimum of three times.

### Phylogenetic relationship of alphasatellite and alpha-rep protein

Nucleotide sequences of 43 full-length alphasatellites species were retrieved from the NCBI GenBank (Additional file 1: Table S1) and their phylogenetic relatedness with CLCuMuA and GDarSLA was generated using the MEGA-X tool [23, 24]. Both CLCuMuA and GDarSLA belongs to the largest genus *Colecusatellite* of the family *Alphasatellitidae* and have a single protein-encoding region α-Rep of 315 amino acids in length. To further decipher the evolutionary pattern of α-Rep protein of CLCuMuA and GDarSLA (19 isolates), a phylogenetic dendrogram was generated by the MEGA-X tool by using maximum-likelihood statistical method, Jonson-Tylor-Thornton (JTT) amino acid substitution model and 1000 bootstrap replicates.

### Genetic variability analysis of alphasatellites

To understand the genetic variability among the cognate (CLCuMuA) and non-cognate (GDarSLA) alphasatellite, 94 full-length nucleotide sequences for GDarSLA and 169 full-length nucleotide sequences for CLCuMuA were selected from the NCBI GenBank (Additional file 1: Table S2). The parameter used for the analysis was; the total number of segregating sites (s), the total number of mutations (η), nucleotide diversity (π), average number of nucleotide differences between sequences (k), estimation of the population mutation rate based on the total number of s-value (θ–w), estimation of the population mutation rate based on the total number of mutations (θ–η), and gene diversity (Hd). These parameters were analysed by the DNA sequence polymorphism tool (DnaSP V 6.12.03) [25, 26].

### Quantitative detection of viral DNAs by real time-PCR (qPCR)

Primers were designed for CLCuMuV-IN, LuLDB, CLCuMuA and GDarSLA using Primer Quest™ tool of Integrated DNA Technologies, Inc. USA (IDT) online software (Additional file 1: Table S3). qPCR reaction was set up using total genomic DNA isolated from infected *N. benthamiana* leaves and SYBR green reaction chemistry. For preparing a standard curve, restriction digestion linearized full-length clone of CLCuMuV-IN, LuLDB, CLCuMuA and GDarSLA were serially diluted ten folds to get a range of 10^10^–10^2^ viral genomic copies per 2 µl plasmid. Plasmid copy number per microliter was calculated and the plasmid dilutions thus obtained was subjected to real-time PCR (Additional file 1: Fig. S1) [27]. Cycling parameters used were as follows: 1 cycle at 50 °C for 2 min, 1 cycle at 95 °C for 3 min (DNA polymerase activation), and 40 cycles, each consisting of 30 s at 95 °C (denaturation) and 20 s at 60 °C (annealing) and 20 s at 72 °C (extension). Dissociation analysis was performed by incubating the reaction at 95 °C for 15 s, annealing at 60 °C for 20 s and 95 °C for 20 min. For each time point and inoculation combination, six samples each with three technical replicates were tested [28, 29]. Statistical significance was calculated using ANOVA and the graphs were made using GraphPad Prism 9 (https://www.graphpad.com/scientific-software/prism/).

## Results

### Sequence and phylogenetic analysis of alphasatellite

**Fig. 2:**
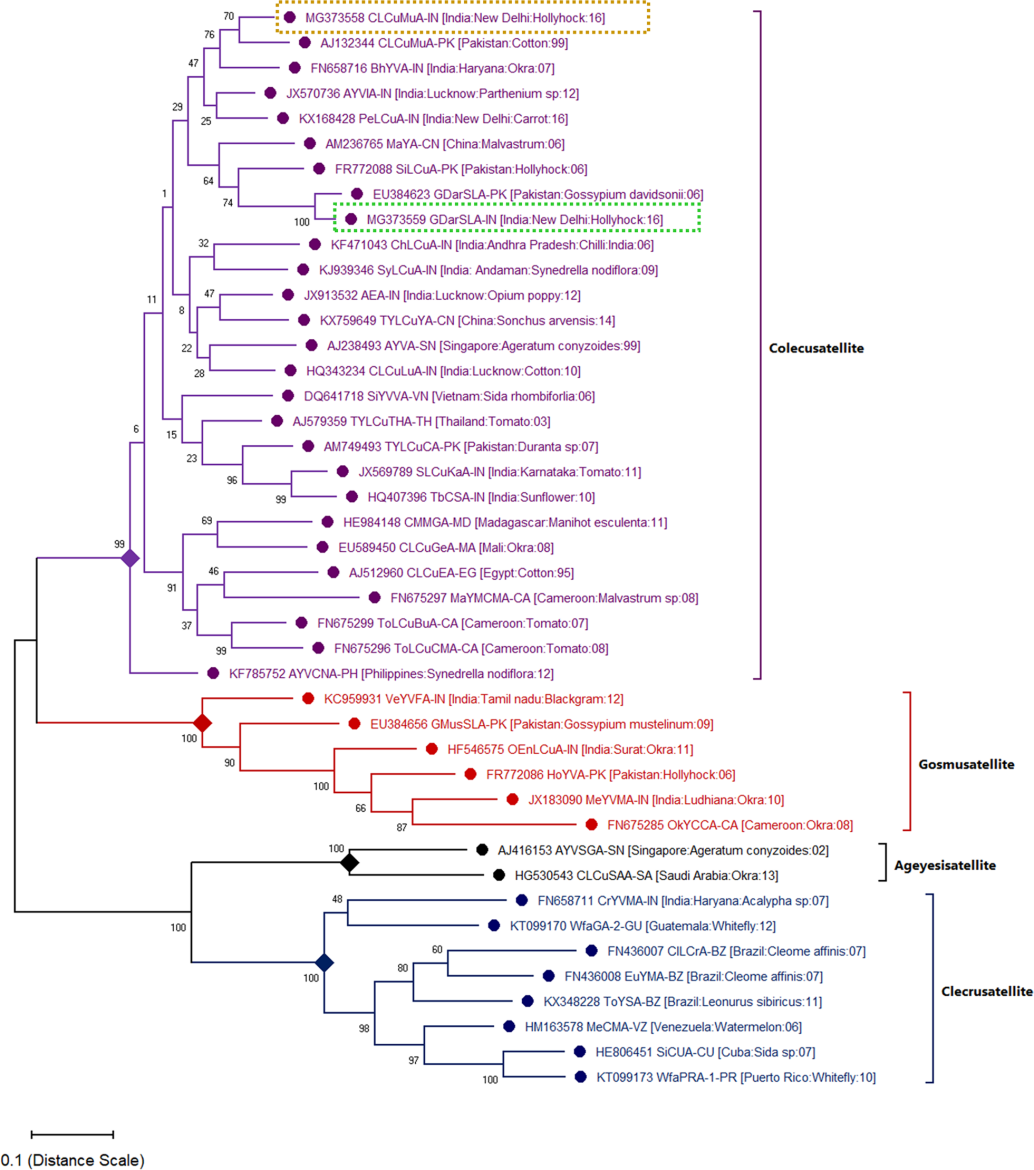
Phylogenetic relationships of *Cotton leaf curl Multan alphasatellite* (CLCuMuA, Accession No. MG373558) and *Gossypium darwinii symptomless alphasatellite* (GDarSLA, Accession No. MG373559) cloned from symptomatic hollyhock plants. Begomovirus associated alphasatellites included in this analysis were selected from NCBIBLAST results and the phylogenetic tree was constructed using the MEGA-X tool. In each case, the database accession number is given. The number at major mode indicates the percentage bootstrap confidence score for 1000 replicates. The distance scale bar denotes the rate of nucleotide substitution per site

**Fig. 3:**
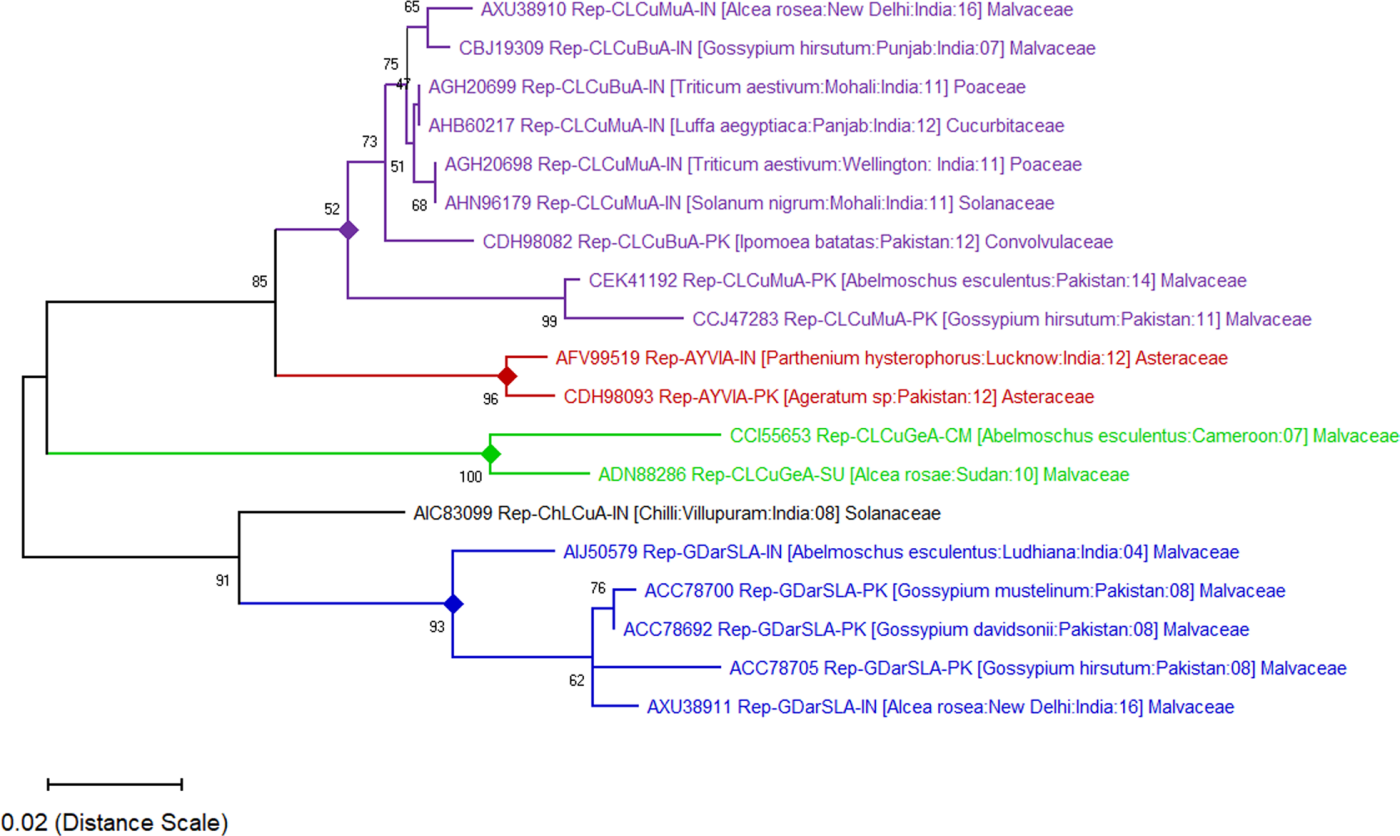
Phylogenetic relatedness of *Cotton leaf curl Multan alphasatellite* (CLCuMuA, Accession no. MG373558) and *Gossypium darwinii symptomless alphasatellite* (GDarSLA, Accession no. MG373559) α-Rep. The α-Rep amino acid sequences from 19 entries in NCBI database were aligned using Maximum-Likelihood statistical method, with Jones–Taylor-Thomson (JTT) amino-acid substitution model, with 1000 bootstrap replicates. The accession number, host, geographical location, year of isolation have been mentioned for identification. The distance scale bar denotes the rate of amino acid substitution per site

Sequence comparison of the two alphasatellites revealed that CLCuMuA (size 1571 bp) and GDarSLA (size 1377 bp) shared 87.53% nucleotide sequence identity. However, the amino acid length of both the α-Rep of satellites was the same (315 aa). The phylogenetic analysis grouped CLCuMuA [India:New Delhi: Hollyhock: 16] with CLCuMuA-PK [Pakistan: Cotton: 99], while GDarSLA [India:New Delhi: Hollyhock: 16] with GDarSLA [Pakistan: *Gossypium davidsonii*: 09]. The two alphasatellites appear to diverge from a close common ancestor (Fig. 2). Alphasatellites encode a single protein, replication initiator protein (α-Rep) and are capable of self-replication. The CLCuMuA are found to be associated with several families of plants such as Malvaceae (*Alcea rosea*, *Gossypium hirsutum*, *Abelmoschus esculentus*), Poaceae (*Triticum aestivum*), Cucurbitaceae (*Luffa aegyptiaca*), Solanaceae (*Solanum nigrum*), and Conolvulaceae (*Ipomoea batatas*) [30–32]. Whereas, GDarSLA has been  majorly reported from the Malvaceae family (*Abelmoschus esculentus, Gossypium hirsutum, Alcea rosea, Gossypium davidsonii, and Gossypium mustelinum*) of plants (Fig. 3) [25, 33]. Evolutionary divergence of cognate alphasatellite α-Rep protein suggests, this alphasatellite infects several families of crop plants. However, non-cognate alphasatellite also infect some non-cultivated host plants such as *G. mustelinum* and *G. davidsonii* apart from the cultivated crop (*A. esculentus*) plants [34].

### Estimation of nucleotide diversity in cognate and non-cognate alphasatellite

To further understand the genetic diversity of CLCuMuA and GDarSLA alphasatellites, gene diversity (Hd) and nucleotide diversity (π) was calculated. The gene diversity (Hd) and nucleotide diversity (π) have been highlighted in bold (Table 1). CLCuMuA have higher π value (π = 0.071) as compare to GDarSLA (π = 0.053) and also have a high degree of gene diversity (Hd = 0.998) in CLCuMuA as compare to GDarSLA (Hd = 0.991). A higher level of genetic diversity in a population is generally linked with the long-term survival of the species and could potentially shape the evolutionary dynamics of a population [35–37].

**Table 1:** Genetic variability analysis of hollyhock-associated alphasatellites

Parameter used^a^	GDarSLA	CLCuMuA
N	94	169
L	1377	1571
s	459	571
η	573	797
π	**0.05345**	**0.07104**
k	56.92052	75.65716
θ–w	0.08567 (Tajima's D: − 1.21539; NS, *P* > 0.10)	0.09257 (Tajima's D: − 0.85623; NS, *P* > 0.10)
θ–η	0.10950 (Tajima's D: − 1.69642; NS, *P* > 0.10)	0.12843 (Tajima's D: − 1.52847; NS, *P* > 0.10)
Hd^b^	0.991** (− 3.45070**)	0.998** (− 3.86968**)

^a^N, total number of sequence analyzed; L, length; s, total number of segregating sites; η, total number of mutations; π, nucleotide diversity for complete genome; k, average number of nucleotide differences between sequences; θ–w, Watterson’s estimate of the population mutation rate based on the total number of segregating sites; θ–η, Watterson’s estimate of the population mutation rate based on the total number of mutations; and Hd, gene diversity

^b^Hd = gene diversity; for GDarSLA = Fu and Li's D* test statistic: − 3.45070; Statistical significance: **, *P* < 0.02; Fu and Li's F* test statistic: − 3.22012; Statistical significance: **, *P* < 0.02 and for CLCuMuA = Fu and Li's D* test statistic: − 3.86968; Statistical significance: **, *P* < 0.02; Fu and Li's F* test statistic: − 3.24863; Statistical significance: **, *P* < 0.02. NS, Non-significant

### Infectivity analysis of the cloned DNA of helper begomovirus and satellites

**Fig. 4:**
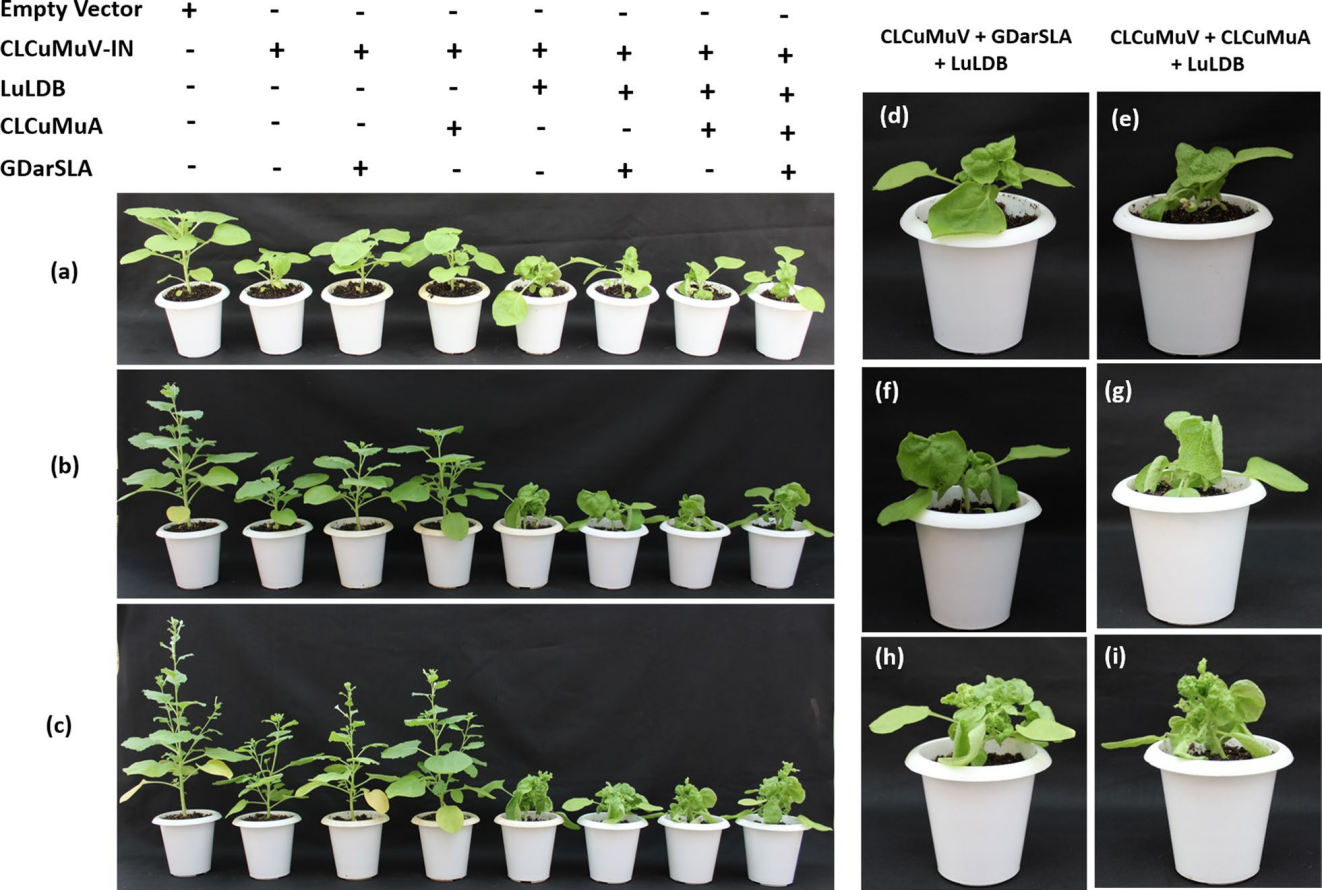
Plant infectivity assay of begomovirus and their associated satellite molecules on *N. benthamiana* plants. **a**–**c** The phenotype of the plants inoculated with either only CLCuMuV or co-agroinoculated with satellite molecules at 7, 14 and 21 dpi, respectively. **d**, **f**, and **g** Close-up photograph of the plant co-agroinoculated with CLCuMuV along with GDarSLA alphasatellite and LuLDB molecules at 7, 14 and 21 dpi, respectively. Similarly, **e**, **g** and **i** enlarged view of the plant co-agroinoculated with CLCuMuV along with CLCuMuA alphasatellite and LuLDB molecules at 7, 14 and 21 dpi, respectively

**Fig. 5:**
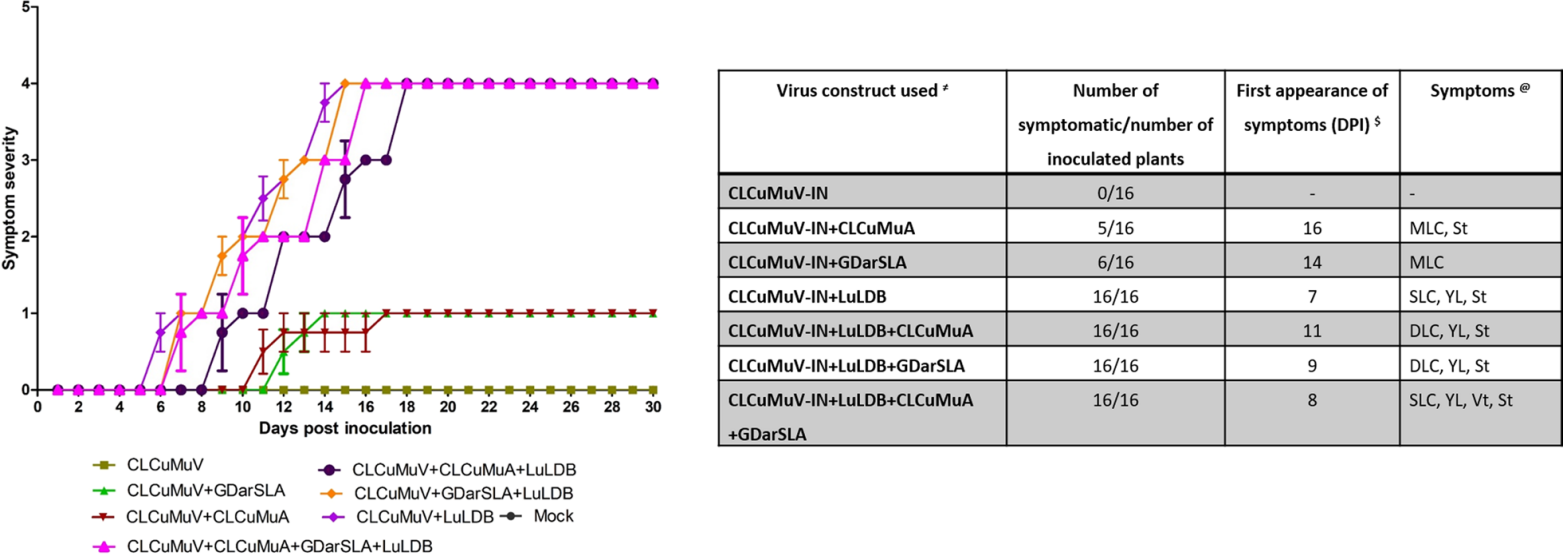
Infectivity analysis of *N. benthamiana* plants inoculated with CLCuMuV-IN, LuLDB, CLCuMuA and GDarSLA in different combinations. The graph represents the symptom severity score (y-axis) vs days post-inoculation (X-axis). The table gives details of the number of symptomatic plants, dpi at which the symptoms were observed and the descriptions of the symptoms observed in each case. @ MLC; mild leaf curling, St; stunted growth, DLC; downward leaf curling, YL; yellowing of leaves, Vt; Vein thickening, SLC; severe leaf curling, ‘–’; no symptoms and $ Day of appearance of first symptoms from the date of inoculation. Symptom severity scale, 0-no symptoms, 1-very mild yellowing, 2-mild yellowing with downward leaf curling, 3-severe yellowing of leaves with downward leaf curling and vein thickening, 4-severe yellowing of leaves with downward leaf curling, vein thickening and stunted plant growth, 5-severe yellowing of leaves with downward leaf curling, vein thickening and stunted plant growth leading to plant death

*N. benthamiana* plants inoculated with an agroinfectious clone of CLCuMuV-IN alone did not develop symptoms. Plants inoculated with CLCuMuV-IN and the dimeric betasatellite LuLDB developed typical symptoms of downward leaf curling, yellowing, vein thickening and stunted growth as early as 7 dpi (Fig. 4a, d, e). In contrast, co-inoculation of CLCuMuV-IN with CLCuMuA or GDarSLA delayed the appearance of the symptom by a week (Fig. 5) and the symptom produced were mild (Fig. 4b, f, g) as compared to plants inoculated with CLCuMuV-IN and LuLDB. Further, a delay of two days was observed in symptom development in plants co-inoculated with GDarSLA and the helper virus as against those co-inoculated with CLCuMuA and the helper virus (Fig. 5). The co-inoculation of betasatellite LuLDB with CLCuMuV-IN and CLCuMuA and/or GDarSLA produced severe symptoms similar to CLCuMuV-IN and LuLDB only combination however, the alphasatellites delayed the appearance of symptoms even in this case (Figs. 4, 5).

### Effect of alphasatellites on helper begomovirus and betasatellite replication

**Fig. 6:**
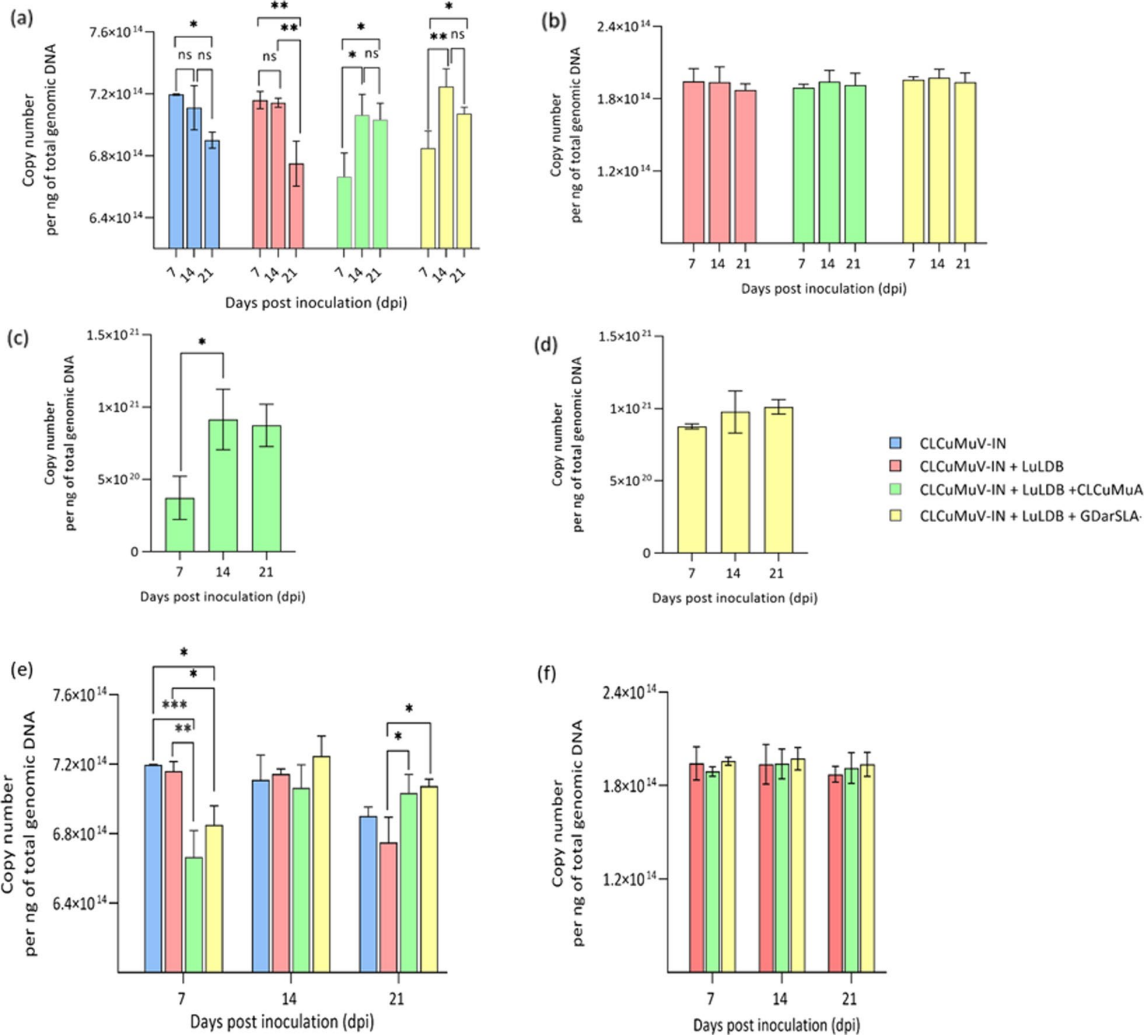
Quantification (qPCR) of viral DNAs in *N. benthamiana* plants inoculated with CLCuMuV-IN, LuLDB, CLCuMuA and GDarSLA in different combinations at 7, 14 and 21 dpi. **a** Quantification of CLCuMuV-IN, **b** Quantification of LuLDB, **c** Quantification of CLCuMuA **d** Quantification of GDarSLA, **e** Comaprison of levels of CLCuMV-IN in *N. benthamiana* plants inoculated with CLCuMuV-IN, LuLDB, CLCuMuA and GDarSLA in different combinations at 7, 14 and 21 dpi. **f** Comaprison of levels of LuLDB in *N. benthamiana* plants inoculated with CLCuMuV-IN, LuLDB, CLCuMuA and GDarSLA in different combinations at 7, 14 and 21 dpi.The graphs represent an average of six samples for each combination. Statistical significance was calculated using ANOVA. *** indicates *p* value 0.0001–0.001, ** indicates *p* value 0.001–0.01, * indicates *p* value 0.01–0.05, ns ≥ 0.05 not significant

As discussed above, the infectivity analysis suggested that the alphasatellites suppress and delay symptom development. To further confirm these observations, we performed quantitative real-time PCR (qPCR) of plants *N. benthamiana* samples inoculated with CLCuMuV-IN and its associated satellite molecules in different combinations at three different time points (Fig. 6a–d). The qPCR results indicate that CLCuMuV-IN accumulates to similar levels in plants inoculated with CLCuMuV-IN alone or together with LuLDB (Fig. 6a). However, in the presence of either alphasatellites, the accumulation of CLCuMuV-IN is significantly reduced at 7 dpi (Fig. 6a, e). But no significant difference in CLCuMuV-IN levels is observed at 14 and 21 dpi (Fig. 6a, e). This observation corroborates the results of infectivity analysis wherein presence of alphasatellite the symptoms appear at 9–11 dpi as against 7 dpi observed in the case of CLCuMuV-IN and LuLDB only combination. Consistent with the infectivity analysis, no difference in CLCuMuV-IN accumulation was observed among the two alphasatellites (Fig. 6a, e). Interestingly, the presence of either alphasatellites does not affect betasatellite accumulation (Fig. 6b, f). LuLDB accumulates to similar levels in plants co-inoculated with CLCuMuV-IN + LuLDB + CLCuMuA or GDarSLA or with CLCuMuV-IN and LuLDB only (Fig. 6b, f).

## Discussion

The association of CLCuMuA alphasatellite in the disease complex might give an advantage for the symptom induction in plants. Only a few geminivirus-associated satellites were reported to be associated with hollyhock plants such as *Cotton leaf curl Bangalore betasatellite* (CLCuBaB) [38], *Cotton leaf curl Multan betasatellite* (CLCuMuB), LuLDB [13]**,** and *Malvastrum yellow mosaic alphasatellite* (MaYA). In recent years, the association of CLCuMuV with Malvaceae (cotton, okra, hollyhock, hibiscus species), Solanaceae (chilli, tomato), Cucurbitaceae (luffa species) family of crop plants have been extensively studied in India and Pakistan (Figs. 2, 3) [39–41]**.** However, the role of satellites in disease-complex has not been elucidated in detail.

The results of this and a previous study [18] confirm that the monopartite begomovirus CLCuMuV-IN, a betasatellite LuLDB and two alphasatellites are associated with yellow mosaic disease of hollyhock. The infectivity analysis and qPCR confirmed that CLCuMuV-IN is capable of replication and systemic infection in *N. benthamiana*. However, symptoms appear only when CLCuMuV-IN and its associated betasatellite, LuLDB are co-inoculated. When either CLCuMuA or GDarSLA was co-inoculated with LuLDB and/or CLCuMuV, symptoms were delayed and attenuated and the helper virus accumulated to relatively lower levels at early stages of infection (Figs. 5, 6e). The alphasatellites did not affect the accumulation of the betasatellite LuLDB (Fig. 6f).

Furthermore, variation in the begomovirus genome allows the virus population to be dynamic and might help broaden their host range [42]. A higher nucleotide diversity index (π =  > 0.07) of CLCuMuA (Table 1) further suggests that this alphasatellite contribute some advantages to the helper virus for replication. However, α-Rep of the CLCuMuA is distinctly related with the α-Rep of GDarSLA and it might modulate the helper virus replication. Previous studies have shown that several alphasatellites are capable of replicating and systemically infecting their host in the presence of a helper virus with varying effects on symptomatology and virulence*. Ageratum yellow vein Singapore alphasatellite* (AYVSGA) reduces symptoms severity and the relative accumulation of its associated betasatellite, *Tomato leaf curl betasatellite* (ToLCB), without affecting the accumulation of the helper virus [15]. In another example, *Tomato yellow leaf curl China alphasatellite* (TYLCCNA) reduces the accumulation of both the helper virus *Tomato yellow leaf curl China virus* (TYLCCNV) and its associated betasatellite *Tomato yellow leaf curl China betasatellite* (TYLCCNB, [16]). Delay and attenuated symptom development and reduction in titer of the helper virus DNA components were also observed when *Ageratum yellow vein alphasatellite* (AYVA) was co-inoculated with different cassava mosaic geminiviruses (CMGs) [43]. Interestingly, a reverse trend was observed in the case of alphasatellite associated WDIV, a mastrevirus. Here, the presence of alphasatellite increased WDIV accumulation and resulted in reduced accumulation of VsiRNA [17]. The contrasting effect of alphasatellites on helper virus and betasatellite accumulation also depends on the virus-host combination. For example, in the presence of *Euphorbia yellow mosaic alphasatellite* (EuYMA), the accumulation of *Euphorbia yellow mosaic virus* (EuYMV) DNA A increases in two plant host *E. heterophylla* and *N. benthamiana,* whereas EuYMA reduces EuYMV DNA-A levels in *Arabidopsis thaliana* [44]*.* On the contrary EuYMV DNA-B accumulation increases in the presence of EuYMA, in *E. heterophylla* and its levels in *N. benthamiana* and *A. thaliana* remains unchanged [44].

Suppression of silencing by α-Rep, its interaction and interference with βC1 activity, blocking the function of the helper virus Rep are some of the hypotheses put forward to explain the mechanism underlying the interaction between alphasatellites and its helper virus/betasatellite complex [13, 15, 45, 46]. Recently, potential genes regulated by TYLCCNA have been identified in a transcriptome profile [16]. Silencing of these TYLCCNA responsive genes causes severe symptoms and increased viral DNA accumulation, suggesting that these genes could contribute to host resistance against TYLCCNV/TYLCCNB infection [16].

## Conclusions

In summary, our results demonstrate a new and differential interaction between alphasatellites CLCuMuA or GDarSLA and the associated helper virus and betasatellite complex. However, the specific molecular mechanism underlying this interaction remains to be determined. The evolutionary dynamics of α-Rep suggest further, it might have evolved from a common ancestor and then undergone selective differentiation and speciation. The higher genetic variability in CLCuMuA alphasatellite suggests that it might have acquired selective advantage during the course of evolution enabling it  to infect several families of crop plants. It has been also hypothesized that alphasatellites associated with begomoviruses might have been acquired by the helper viruses to modulate the virulence to achieved enhance virus fitness. As these interactions could be host-specific, the interaction between these viral DNAs in the natural host hollyhock remains to be determined. Based on this study, transcriptome analysis of the differentially regulated genes in plants co-inoculated with alphasatellites, the nature of the interaction of α-Rep with other viral proteins such as helper viral Rep and Transcriptional activation protein (TrAP) and cellular replisome can be examined to get molecular details of these interactions. Alpha-Rep is known to suppress TGS which could reduce viral DNA methylation allowing replication. It is tempting to speculate that α-Rep could modulate other epigenetic modifications by interacting with various cellular and viral proteins, which could, in turn, affect replication and accumulation of the helper virus. These studies could help us understand why satellite DNA molecules are acquired by the helper viruses and the biological significance of these interactions.

## Supplementary Information

**Additional file 1.** Details of the sequences, primers and standard curves of viral DNAs used for the phylogenetic analysis and quantitative real-time PCR.

## Data Availability

All data generated or analysed during this study are included in this published article and supplementary information files. The genome sequences of the geminiviruses, betasatellite and alphasatellites are available at the GenBank (https://www.ncbi.nlm.nih.gov/genbank/).

## Abbreviations

CLCuMuV-IN: Cotton leaf curl Multan virus-India
CLCuMuA: Cotton leaf curl Multan alphasatellite
GDarSLA: Gossypium darwinii symptomless alphasatellite
LuLDB: Ludwigia leaf distortion betasatellite
CAGs: Cassava-begomoviruses
MEGA-X tool: Molecular evolutionary genetic analysis tool
PTR: Partial tandem repeat constructs
qPCR: Quantitative PCR
PTGS: Post-transcriptional gene silencing
TGS: Transcriptional gene silencing
JTT: Jonson–Tylor-Thornton
SCR: Satellite conserved region
Ng: Nanogram
