# Supplementary material for: Roles of two distinct alphasatellites modulating geminivirus pathogenesis
Source: Virol J. 2021 Dec 13;18:249. doi: 10.1186/s12985-021-01718-6 (PMC8670188; doi:10.1186/s12985-021-01718-6)
Supplement: Supplementary file 1 — Additional file 1. Details of the sequences, primers and standard curves of viral DNAs used for the phylogenetic analysis and quantitative real-time PCR. [file 12985_2021_1718_MOESM1_ESM.docx]

**Additional file 1-Details of the nucleotide sequences, primers and standard curve for viral DNAs used for the phylogenetic analysis and quantitative real-time PCR.**

Table S1: List of geminiviruses-associated alphasatellites used in this study.

| **Alphasatellite** | **Accession no.** | **Abbreviation used** |
| --- | --- | --- |
| Ageratum yellow vein Singapore alphasatellite | AJ416153 | AYVSGA |
| Cotton leaf curl Saudi Arabia alphasatellite | HG530543 | CLCuSAA |
| Cleome leaf crumple alphasatellite | FN436007 | ClLCrA |
| Croton yellow vein mosaic alphasatellite | FN658711 | CrYVMA |
| Euphorbia yellow mosaic alphasatellite | FN436008 | EuYMA |
| Melon chlorotic mosaic alphasatellite | HM163578 | MeCMA |
| Sida Cuba alphasatellite | HE806451 | SiCUA |
| Tomato yellow spot alphasatellite | KX348228 | ToYSA |
| Whitefly associated Guatemala alphasatellite 2 | KT099170 | WfaGA 2 |
| Whitefly associated Puerto Rico alphasatellite 1 | KT099173 | WfaPRA 1 |
| Ageratum enation alphasatellite | JX913532 | AEA |
| Ageratum yellow vein alphasatellite | AJ238493 | AYVA |
| Ageratum yellow vein China alphasatellite | KF785752 | AYVCNA |
| Ageratum yellow vein India alphasatellite | JX570736 | AYVIA |
| Bhendi yellow vein alphasatellite | FN658716 | BhYVA |
| Cassava mosaic Madagascar alphasatellite | HE984148 | CMMGA |
| Chilli leaf curl alphasatellite | KF471043 | ChLCuA |
| Cotton leaf curl Egypt alphasatellite | AJ512960 | CLCuEA |
| Cotton leaf curl Gezira alphasatellite | EU589450 | CLCuGeA |
| Cotton leaf curl Lucknow alphasatellite | HQ343234 | CLCuLuA |
| Cotton leaf curl Multan alphasatellite | AJ132344 | CLCuMuA |
| Gossypium darwinii symptomless alphasatellite | EU384623 | GDarSLA |
| Malvastrum yellow mosaic alphasatellite | AM236765 | MaYA |
| Malvastrum yellow mosaic Cameroon alphasatellite | FN675297 | MaYMCMA |
| Pedilanthus leaf curl alphasatellite | KX168428 | PeLCuA |
| Sida leaf curl alphasatellite | FR772088 | SiLCuA |
| Sida yellow vein Vietnam alphasatellite | DQ641718 | SiYVVA |
| Sunflower leaf curl Karnataka alphasatellite | JX569789 | SLCuKaA |
| Synedrella leaf curl alphasatellite | KJ939346 | SyLCuA |
| Tobacco curly shoot alphasatellite | HQ407396 | TbCSA |
| Tomato leaf curl Buea alphasatellite | FN675299 | ToLCuBuA |
| Tomato leaf curl Cameroon alphasatellite | FN675296 | ToLCuCMA |
| Tomato yellow leaf curl China alphasatellite | AM749493 | TYLCuCA |
| Tomato yellow leaf curl Thailand alphasatellite | AJ579359 | TYLCuTHA |
| Tomato yellow leaf curl Yunnan alphasatellite | KX759649 | TYLCuYA |
| Gossypium mustelinum symptomless alphasatellite | EU384656 | GMusSLA |
| Hollyhock yellow vein alphasatellite | FR772086 | HoYVA |
| Mesta yellow vein mosaic alphasatellite | JX183090 | MeYVMA |
| Okra enation leaf curl alphasatellite | HF546575 | OEnLCuA |
| Okra yellow crinkle Cameroon alphasatellite | FN675285 | OkYCCA |
| Vernonia yellow vein Fujian alphasatellite | KC959931 | VeYVFA |
| Cotton leaf curl Multan alphasatellite | MG373558 | CLCuMuA |
| Gossopium darwinii symptomless alphasatellite | MG373559 | GDarSLA |

Table S2: List of CLCuMuA and GDarSLA used for the genetic variability analysis.

| **Alphasatellite molecules** | **NCBI GenBank Accession no.** |
| --- | --- |
| Cotton leaf curl Multan alphasatellite (CLCuMuA) | MT037048, MT037047, MT037046, MW166936, MW166935, MW166934, MW414530, MT966814, MT966813, MT966812, MK357298, MK357297, MK357296, MK357295, MK357294, MK357293, MK357292, MK357291, MK357290, MK357289, MK357288, MK357287, MN922310, MK357286, LT840042, LT840041, LT840040, LT840039, LT840038, LT840037, LT840047, LT840046, LT840045, LT840043, MG735452, MG735451, MF289498, MH450227, MH517042, MH510287, MH510286, MH510285, MH510284, MH510283, MH510282, MH510281, MH510280, MH510279, MG373554, LT608337, MH252995, MF344548, MF344547, MF344546, MF141740, MF141735, MF141734, MF141733, MF141732, LN886537, LN870408, LN870407, LN870406, LN870405, LN870404, LN870403, LN870402, LN870401, LN870400, LN870399, LN870398, LN870397, LN870396, LN870395, LN870394, LN870393, LN870392, LN870390, LN870389, LN870388, LN870387, LN870386, LN866264, KX656851, KX656846, KX656845, KX656844, KX656843, LN831972, LN831971, LN831970, LN831969, LN831968, LN831967, LN831966, LN829153, LN829152, LN829147, LN829146, LN829145, LN829144, LN829143, LN829142, LN829141, LN829140, LN829139, LN829138, LN829137, LN829136, LN829135, LN829134, LN829133, LN829132, LN829131, LN829130, KY783480, LN810541, LN874310, LN874309, LN874307, LN874306, LN874302, LN874301, LN874300, LN874299, LN874298, LN874297, LN874296, LN874295, LN874294, HG934819, HG934817, HG934818, LN713498, LN713496, LN713495, LN713494, LN713493, LN713492, LN713488, LN713487, LN713486, LN713485, LN713484, LN713483, LN713482, LT615045, KR816015, KR816014, KR816013, KR816011, KR816010, KR816009, KR816008, HG934391, KR816017, KR816016, HF564605, HF564602, HF564601, HF564600, HE979548, HE979546, HE978348, HE978347, HE978346, HE978345, HE966424, HE965684, HE965680, KJ028212, KC305093, FR873573, FR873572, FR873571, HE599399, HE599398, FR877532 |
| Gossopium darwinii symptomless alphasatellite (GDarSLA) | LT615046, MT037045, MT037044, MT037043, MT966817, MT966816, MT966815, MH760464, MH760460, MH817849, MH517039, MH517038, MH517037, MH517036, MH517035, MH517034, MH517032, MG373559, MF683830, LT549471, MF141744, MF141743, MF141742, MF141741, MF141738, MF929032, MF929023, KX656852, KX656842, KX656841, KX656840, KX656839, KX656838, KX656836, LN829158, LN713497, LN811058, LN811057, LN874305, LN874303, HG934799, HG530128, KT390426, HE972276, HE965679, HE965678, KM103525, FR877536, FR877533, FR772092, FR772084, EU384626, EU384651, EU384650, EU384649, EU384648, EU384647, EU384646, EU384645, EU384643, EU384642, EU384641, EU384640, EU384639, EU384638, EU384637, EU384636, EU384634, EU384633, EU384632, EU384630, EU384629, EU384628, EU384627, EU384625, EU384624, EU384623, EU384622, EU384621, EU384620, EU384619, EU384618, EU384617, EU384616, EU384615, EU384614, EU384613, EU384612, EU384611, EU384610, EU384609, EU384607, EU384606, FJ218493 |

Table S3: List of primers used for the qRT-PCR

| **Viral Molecules** | **Primers** | **Primers Sequences** | **Tm**  **(^ο^C)** | **Percent GC content.**  **(%)** | **Length of the primer**  **(bp)** |
| --- | --- | --- | --- | --- | --- |
| (CLCuMuA, Accession no. MG373558) and (GDarSLA, Accession no. MG373559) | V904qRT-F | CGAAGCTCGGGATTACTGTATG  Amplicon-143bp | 62 | 50 | 22 |
|  | V905qRT-R | GATTCTCCTCAGCCATTCTCAC | 62 | 50 | 22 |
| *Cotton leaf curl Multan Virus* (CLCuMuA)  Accession no MG373551 | V918qRT-F | GCACATTTCCATCCGAACATAC  Amplicon-200bp | 62 | 45.5 | 22 |
|  | V918qRT-R | TTAATGACTCTAAGAGCCTCTGATT | 62 | 50 | 22 |
| *Ludwigia leaf distortion betasatellite* (LuLDB)  Accession no: MG373553 | V931qRT-F | GACAAGGAGCGGAAGAAACA  Amplicon-112bp | 62 | 45.5 | 20 |
|  | V931qRT-R | CGATGGTGACATGGTGGATAC | 62 | 50 | 21 |


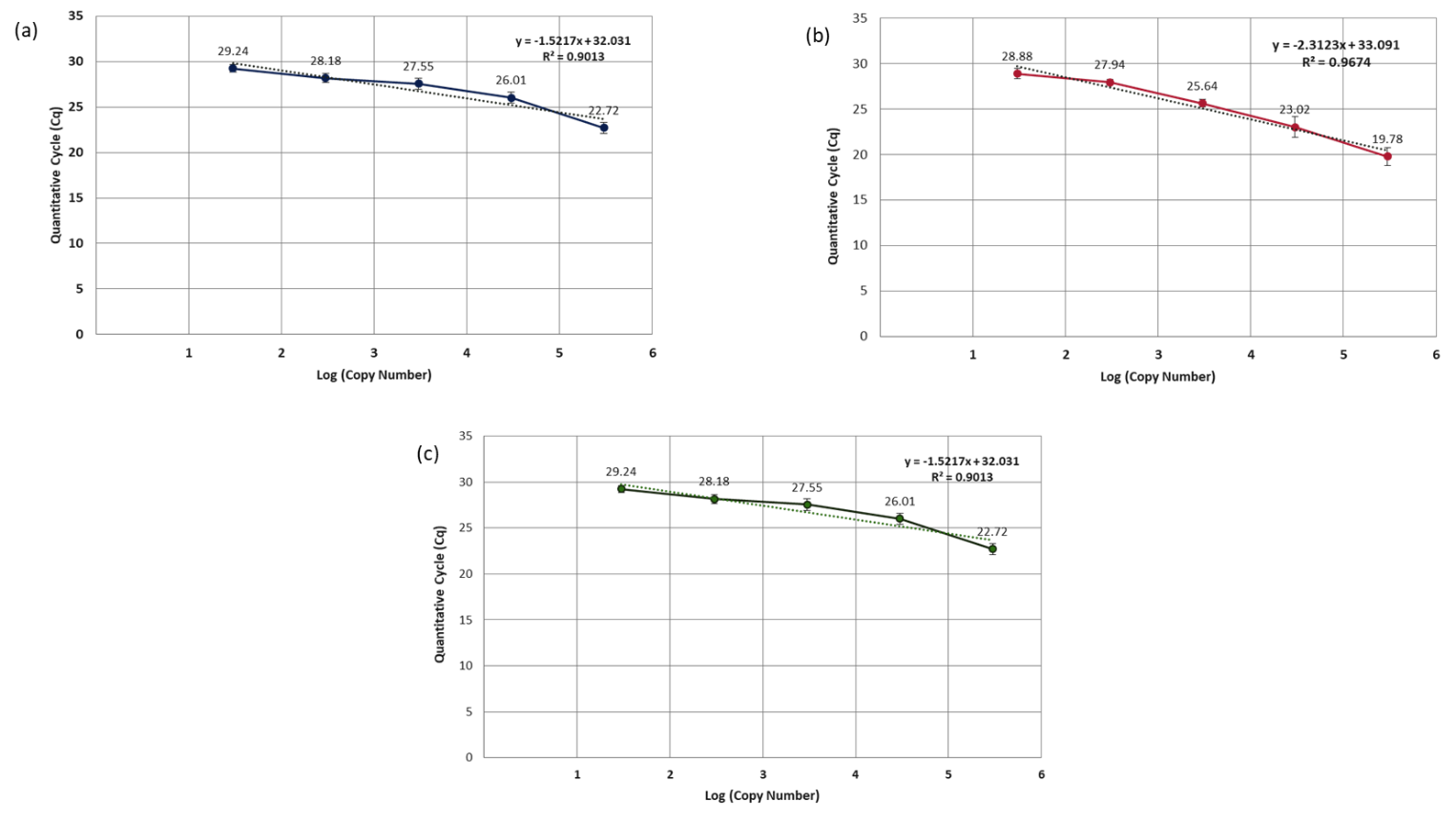


Fig. S1: Standard curve for (a) CLCuMuV-IN, (b) LuLDB and (c) CLCuMuA-IN. A linear regression curve was generated by plotting the CT-values on the X-axis vs. log of the staring quantity (number of copies of the plasmid DNA) on Y-axis. The experiment was performed in triplicate. The equation of the straight line and the coefficient of correlation (R2) are represented on the graph.
